# Therapeutic Conflicts in Emergency Department Patients with Multimorbidity: A Cross-Sectional Study

**DOI:** 10.1371/journal.pone.0110309

**Published:** 2014-10-13

**Authors:** Stefan Markun, Barbara M. Holzer, Roksana Rodak, Vladimir Kaplan, Claudia C. Wagner, Edouard Battegay, Lukas Zimmerli

**Affiliations:** 1 Division of Internal Medicine, University Hospital Zurich, Zurich, Switzerland; 2 Institute of General Practice and Health Service Research, University of Zurich, University Hospital of Zurich, Zurich, Switzerland; 3 Center of Competence Multimorbidity, University of Zurich, Zurich, Switzerland; 4 University Research Priority Program Dynamics of Healthy Aging, University of Zurich, Zurich, Switzerland; French National Centre for Scientific Research, France

## Abstract

**Background:**

Patients with multimorbidity are an increasing concern in healthcare. Clinical practice guidelines, however, do not take into account potential therapeutic conflicts caused by co-occurring medical conditions. This makes therapeutic decisions complex, especially in emergency situations.

**Objective:**

The aim of this study was to identify and quantify therapeutic conflicts in emergency department patients with multimorbidity.

**Methods:**

We reviewed electronic records of all patients ≥18 years with two or more concurrent active medical conditions, admitted from the emergency department to the hospital ward of the University Hospital Zurich in January 2009. We cross-tabulated all active diagnoses with treatments recommended by guidelines for each diagnosis. Then, we identified potential therapeutic conflicts and classified them as either major or minor conflicts according to their clinical significance.

**Results:**

166 emergency inpatients with multimorbidity were included. The mean number of active diagnoses per patient was 6.6 (SD±3.4). We identified a total of 239 therapeutic conflicts in 49% of the of the study population. In 29% of the study population major therapeutic conflicts, in 41% of the patients minor therapeutic conflicts occurred.

**Conclusions:**

Therapeutic conflicts are common among multimorbid patients, with one out of two experiencing minor, and one out of three experiencing major therapeutic conflicts. Clinical practice guidelines need to address frequent therapeutic conflicts in patients with co-morbid medical conditions.

## Introduction

Internationally comparable data with respect to the numbers of patients with two or more concurrent medical conditions are scarce because of heterogeneous definitions of multimorbidity. [1], [2] However, the number of patients with multimorbidity is increasing, particularly among older adults. Almost two thirds of all Americans older than 65 years suffer from multimorbidity. A similar percentage (62%) has been reported from Germany. [3]–[5] This makes managing the care of such patients challenging, especially in emergency situations when physicians see the patient for the first time and make quick decisions regarding appropriate therapy. In such situations, evidence-based treatment guidelines designed for single diseases can lead to serious therapeutic conflicts and cannot be relied upon to provide guidance.

The process of systematically generating information about how to provide appropriate medical support for specific diseases through randomized controlled trials and then consolidating the information in the form of generally applicable treatment strategies known as clinical practice guidelines fails in some notable respects. Multimorbid patients are frequently underrepresented or even systematically excluded from evidence-generating studies [6]–[9], thus limiting the applicability of the guidelines. In addition, potentially adverse drug-drug interactions or highly complex or even inadequate drug regimens may pose problems. [10]–[13] When the recommended therapy for treating one disease is contraindicated in the presence of another concurrent medical condition, this further limits the usefulness of clinical practice guidelines. To our knowledge, there are as yet no estimates of the burden of such therapeutic conflicts in emergency departments.

Therefore, the aim of this study was to focus on identifying and quantifying therapeutic conflicts in cases where emergency department patients had been diagnosed with two or more concurrent medical conditions, and then to characterize the identified potential therapeutic conflicts with respect to their clinical relevance and severity.

## Methods

We designed this study as a retrospective study to estimate the potential magnitude and scope of the problem within the context of care provided in an emergency department.

The setting is an university teaching hospital that provides primary, secondary, and tertiary care for a region with a population of approximately 400,000. The hospital’s emergency department treats about 36,000 patients annually (including surgical patients), about 80% of whom are treated on an outpatient basis.

All consecutive non-surgical cases entered in the emergency department registered from January 1 through January 31, 2009, were screened for eligibility. To be included in the study, patients had to be 18 years of age or older and had to be admitted to a medical ward subsequent to their admission to the emergency department. Patients were excluded from this population if they were not multimorbid, their cases were managed by a multidisciplinary team (for example, patient care managed in the resuscitation room) or if their medical documentation was incomplete. Patient information was anonymized and de-identified prior to analysis.

The study was approved by the local ethics committee (“Kantonale Ethikkommission Zürich”, www.kek.zh.ch, reference number KEK-ZH-NR: 2010-0166/1).

### Definitions

*Active diagnosis* was defined as a medical condition that required diagnostic or therapeutic attention both during the time the patient was in the emergency department and during the time the patient was subsequently hospitalized on a medical ward.

*Treatment recommendation* was defined as a specific diagnostic or therapeutic intervention that is recommended by clinical practice guidelines for a particular diagnosis. This could include the use of drugs (including contrast agents) or non-pharmacological interventions (e.g., pleurocentesis or compression stockings).

*Major therapeutic conflict* was defined as a situation where clinical practice guidelines recommend a treatment of one medical condition that is absolutely contraindicated because of a co-existing condition (for example, a situation where anticoagulation is recommended because of a pulmonary embolism, but at the same time contraindicated because of a co-existing gastrointestinal bleeding).

*Minor therapeutic conflict* was defined as a where clinical practice guidelines recommend a treatment of one medical condition that is relatively contraindicated because of a co-existing condition (for example, a situation where acetylsalicylic acid is recommended because of a vascular disease, but at the same time contraindicated because of a co-existing reflux esophagitis), but where the treatment is possible without adverse effects if certain precautions are taken.

*No conflict* was defined as a medical situation in which a potential therapeutic conflict could be resolved by choosing an equally effective alternative treatment for one of the medical conditions, thus avoiding the conflict with respect to treatment regimens. For example, diabetes in conjunction with severe renal failure was not identified as posing a therapeutic conflict because the treatment for diabetes, metformin, could be replaced with the administration of insulin.

### Data collection and analysis

Data extracted from the hospital’s electronic clinical information system and transferred into Microsoft Excel spreadsheets included the patient’s identification number, case number, date of admission to the hospital, gender, age, and all active diagnoses at the time of admission to the emergency department. Physicians’ notes, laboratory results, and medication orders available at the time of a patient’s admission to the emergency department were scanned for each patient. Because there is no standardized method for identifying and classifying therapeutic conflicts, we followed a pragmatic method of manually classifying therapeutic conflicts through consensus by two experienced clinicians. A four-step process was set up for investigating potential therapeutic conflicts to ensure that definitions of medical conditions, therapies, and treatment conflicts were applied correctly and consistently throughout the selection and evaluation process.

#### Step 1: Creating a list of active diagnoses and associated clinical practice guidelines

Two medical residents each created a comprehensive list of the active diagnoses for each patient. A diagnosis that was not mentioned explicitly in the initial admission report was included if, on the basis of laboratory results, the medical condition was obviously present. The following diagnoses were deduced from laboratory results: anemia (hemoglobin <13.4 g/dL in male patients or <11.7 g/dL in female patients), thrombocytopenia (platelets <143 G/L), neutropenia (neutrophilic granulocytes <1.40 G/L), and renal failure (estimated GFR [glomerular filtration rate] <60 mL/min from MDRD [Modification of Diet in Renal Disease Study Group] equation). [14] Diagnoses were also deduced from prescriptions for medications only if a plausible and unique indication for the medication being prescribed was apparent. No active diagnoses were assumed from prescriptions for medications whose use could be prescribed for any one of several different conditions.

Each resident then classified diagnoses according to the International Classification of Diseases (ICD-10) [15], with the addition of codes to denote therapeutic strategies if appropriate (for example, specifying whether diabetes type 2 was insulin dependent or was being treated with an oral antidiabetic medication). Quality control assurance in this step was according to methods specified in Gilbert et al. [16]. Differences in the two residents’ interpretations or applications of codes were discussed in periodic meetings with senior staff physicians in order to improve coding rules and thus improve uniformity consistency and reproducibility in the data acquisition process.

Information about appropriate treatment recommendations for the identified medical conditions came from relevant clinical practice guidelines available in the evidence-based electronic textbook UpToDate [17] without reference to any other co-existing medical condition.

#### Step 2: Cross-tabulating active diagnoses and treatment recommendations

The medical residents cross-tabulated all active diagnoses with their corresponding evidence-based treatment recommendations for each patient

#### Step 3: Screening for therapeutic conflicts

The medical residents systematically and independently assessed all cases in terms of the applicability of the treatment recommendations in each case, taking into account all active diagnoses for co-occurring medical conditions. Any therapeutic conflicts that they found were characterized with respect to the type of therapy and the type of contraindicating medical condition that was involved. Each therapeutic regimen associated with an identified therapeutic conflict was coded to enable counting of coincidences with the ICD-10 codes of the conflicting co-existing medical conditions. Their separate assessments were compared and any differences resolved in meetings with senior staff physicians.

#### Step 4: Classifying therapeutic conflicts

Senior staff physicians classified each therapeutic conflict as either a major or a minor conflict depending on the clinical importance and the severity of the conflict. Differences in assessments were resolved by consensus.

### Statistical analyses

Continuous data are presented as mean and standard deviation. Categorical data are presented as counts and proportions. Confidence intervals for counts were based on the Poisson distribution.

Microsoft Excel (Microsoft, WA, USA) and STATA (College Station, TX, USA) were used for all statistical analyses.

## Results

During the month of the study, 1520 medical patients were treated in the emergency department of the hospital. Of these cases, 1354 patients were excluded from the study because 1196 had been treated on an outpatient basis or were not mutlimorbid, 2 were under 18 years of age, the care of 130 had been managed by a multidisciplinary medical team, and medical documentation for 26 was incomplete.

**Figure 1:**
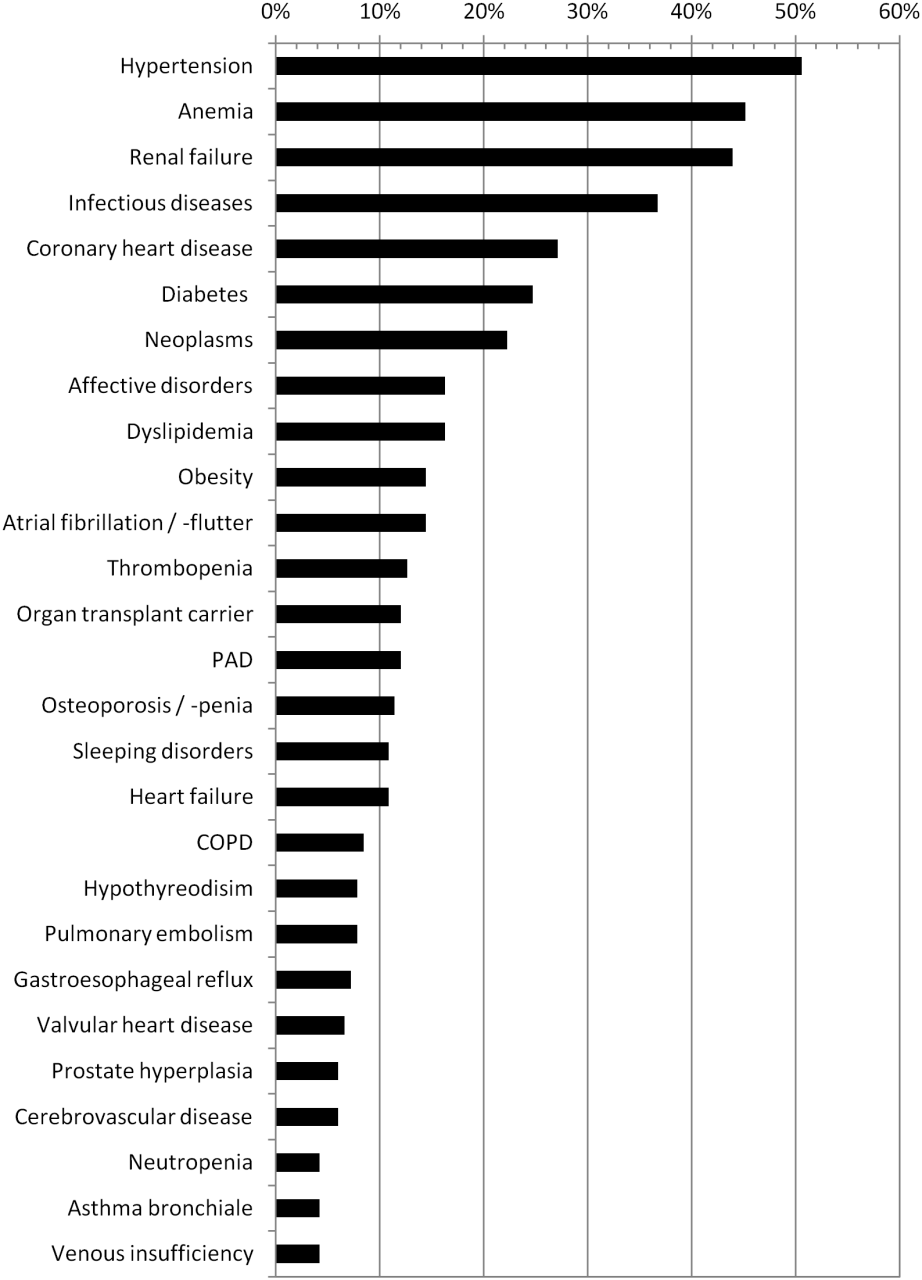
Prevalence of the most frequently diagnosed medical conditions of 166 emergency patients with multimorbity*. *Only those medical conditions diagnosed in ≥4% of the study population are shown PAD = peripheral artery disease; COPD = chronic obstructive pulmonary disease.

Of the remaining 166 patients who were included in the study, 98 (59%) were male (Table 1). The mean age of all patients was 62.7 (SD±19) years, their duration of hospitalisation was on average 12.6 days (SD±21.1).The mean number of active diagnoses per patient was 6.6 (SD±3.4) (range 2–16). About 40% of the patients had eight or more active diagnoses, and almost 14% had eleven or more active diagnoses. The most frequently diagnosed medical conditions in these patients were hypertension (51%), anemia (45%), and renal failure (44%). The prevalence of the most frequently encountered medical conditions is shown in Figure 1.

**Table 1:** Patient characteristics of therapeutic conflicts in 166 emergency patients with multimorbity.

Variable	Number of patients	Percentage of study population
Gender		
Female	68	41.0%
Male	98	59.0%
Age (years)		
<40	24	14.5%
40 to 59	34	20.5%
60 to 79	76	45.8%
>80	32	19.3%
Mode of admission:		
Physician	62	37.3%
Self-referred	53	31.9%
Ambulance	46	27.7%
Others	5	3.0%
Number of active diagnoses per patient:		
2–4	53	31.9%
5–7	46	27.7%
8–10	44	26.5%
≥11	23	13.9%
Duration of hospitalization (days):		
1–3 days	47	28.3%
4–7 days	39	23.5%
8–14 days	40	24.1%
≥14 days	40	24.1%
Patients without therapeutic conflicts	84	50.6%
Patients with therapeutic conflicts:	82	49.4%
Patients with major conflicts only	14	8.4%
Patients with minor conflicts only	34	20.5%
Patients with both major and minor conflicts	34	20.5%

Therapeutic conflicts were identified in 82 (49.4%) of the patients. Major therapeutic conflicts were identified in 28.9% of all patients, minor therapeutic conflicts in 41.0% of all patients. Of the total 239 therapeutic conflicts, 66 (27.6%) were major therapeutic conflicts. The most frequently encountered major conflict was between immunosuppressive therapy (mostly associated with organ transplant recipients) and a co-occurring infectious disease. This situation existed in 10.8% of all cases. Table 2 depicts a complete list of all identified major therapeutic conflicts. The most frequently encountered minor therapeutic conflict, occurring in 13.9% of all patients, was between diuretic therapy (primarily for heart failure) and a co-occurring severe chronic or acute renal failure or renal failure of undetermined origin that required close renal and hemodynamic monitoring. In table 3 a list of identified minor therapeutic conflicts is presented.

**Table 2:** Major therapeutic conflicts identified in 166 emergency patients with multimorbity*.

Type of therapy recommended by CPG	Medical conflict	Number of conflicts	Percentage of study population
Chemotherapy		22	
	Infection	8	4.8%
	Aplasia; neutropenia†	4	2.4%
	Thrombocytes <50 G/L	4	2.4%
	Gastroenteritis	3	1.8%
	Gastrointestinal bleeding	1	0.6%
	Renal failure‡	1	0.6%
	Hemoglobin <6 g/dL	1	0.6%
Immunosuppression		21	
	Infection	18	10.8%
	Aplasia; neutropenia†	3	1.8%
Acetylsalicylic acid		9	
	Gastrointestinal bleeding	4	2.4%
	Acetylsalicylic acid allergy	3	1.8%
	Subdural hemorrhage	1	0.6%
	INR >6	1	0.6%
Contrast agent		3	
	Renal failure‡	2	1.2%
	Contrast dye allergy	1	0.6%
Heparin		2	
	Gastrointestinal bleeding	1	0.6%
	Thrombocytes <50 G/L	1	0.6%
Antihypertensive agents		2	
	Orthostatic dysregulation	2	1.2%
Fluid replacement		2	
	Acute congestive heart failure	2	1.2%
Estrogen replacement		2	
	Acute coronary syndrome	2	1.2%
Oral anticoagulant		1	
	Gastrointestinal bleeding	1	0.6%
Pleurocentesis		1	
	Thrombocytes <50 G/L	1	0.6%
Ganciclovir		1	
	Aplasia; neutropenia†	1	0.6%

CPG = Clinical practice guideline.

INR = international normalized ratio index of blood coagulability.

*Major therapeutic conflict was defined as a situation where clinical practice guidelines recommend a treatment of one medical condition that is absolutely contraindicated because of a co-existing condition.

† Neutropenia defined as neutrophilic granulocytes <1.40 G/L.

‡ Renal failure defined as an estimated GFR [glomerular filtration rate] <60 mL/min from MDRD [Modification of Diet in Renal Disease Study Group] equation.

**Table 3:** Minor therapeutic conflicts identified in 166 emergency patients with multimorbity*.

Type of therapy recommended by CPG	Medical conflict	Number of conflicts	Percentage of study population
Steroid		53	
	Arterial hypertension	16	9.6%
	Diabetes mellitus	13	7.8%
	Osteoporosis	9	5.4%
	Affective disorder	5	3.0%
	Sleeping disorder	5	3.0%
	Obesity	3	1.8%
	Acute congestive heart failure	2	1.2%
Diuretic		28	
	Renal failure†	23	13.9%
	Infection	3	1.8%
	Hyperparathyroidism	2	1.2%
Antihypertensive agents		26	
	PAD	15	9.0%
	Gastrointestinal bleeding	8	4.8%
	Sepsis	3	1.8%
Immunosuppression		19	
	Renal failure†	15	9.0%
	Gastroenteritis	2	1.2%
	Carrier of multiresistant bacteria	1	0.6%
	Thrombocytes <50 G/L	1	0.6%
Beta-blocker		12	
	PAD	4	2.4%
	Acute congestive heart failure	4	2.4%
	Heart block (first-degree)	2	1.2%
	Asthma	2	1.2%
Chemotherapy		10	
	Renal failure†	7	4.2%
	Esophagitis or GERD	2	1.2%
	Skin lesion	1	0.6%
Aspirin		9	
	Esophagitis or GERD	8	4.8%
	Peptic ulcer	1	0.6%
Opioid		5	
	COPD	2	1.2%
	Prostate hyperplasia	2	1.2%
	Constipation	1	0.6%
Compression stockings		2	
	PAD	2	1.2%
Fluid replacement		2	
	Mitral insufficiency	2	1.2%
Estrogen replacement		2	
	Arterial hypertension	2	1.2%
Oxygen		2	
	COPD	2	1.2%
Benzodiazepine		1	
	Encephalopathy	1	0.6%
NSAID		1	
	Esophagitis or GERD	1	0.6%
ACE inhibitor		1	
	Hyperpotassemia	1	0.6%

CPG = Clinical practice guideline.

PAD = peripheral artery disease; GERD = gastroesophageal reflux disease; COPD = chronic obstructive pulmonary disease; ACE = angiotensin-converting enzyme; NSAID = nonsteroidal anti-inflammatory drug.

*Minor therapeutic conflict was defined as a where clinical practice guidelines recommend a treatment of one medical condition that is relatively contraindicated because of a co-existing condition, but where the treatment is possible without adverse effects if certain precautions are taken.

† Renal failure defined as an estimated GFR [glomerular filtration rate] <60 mL/min from MDRD [Modification of Diet in Renal Disease Study Group] equation.

**Figure 2:**
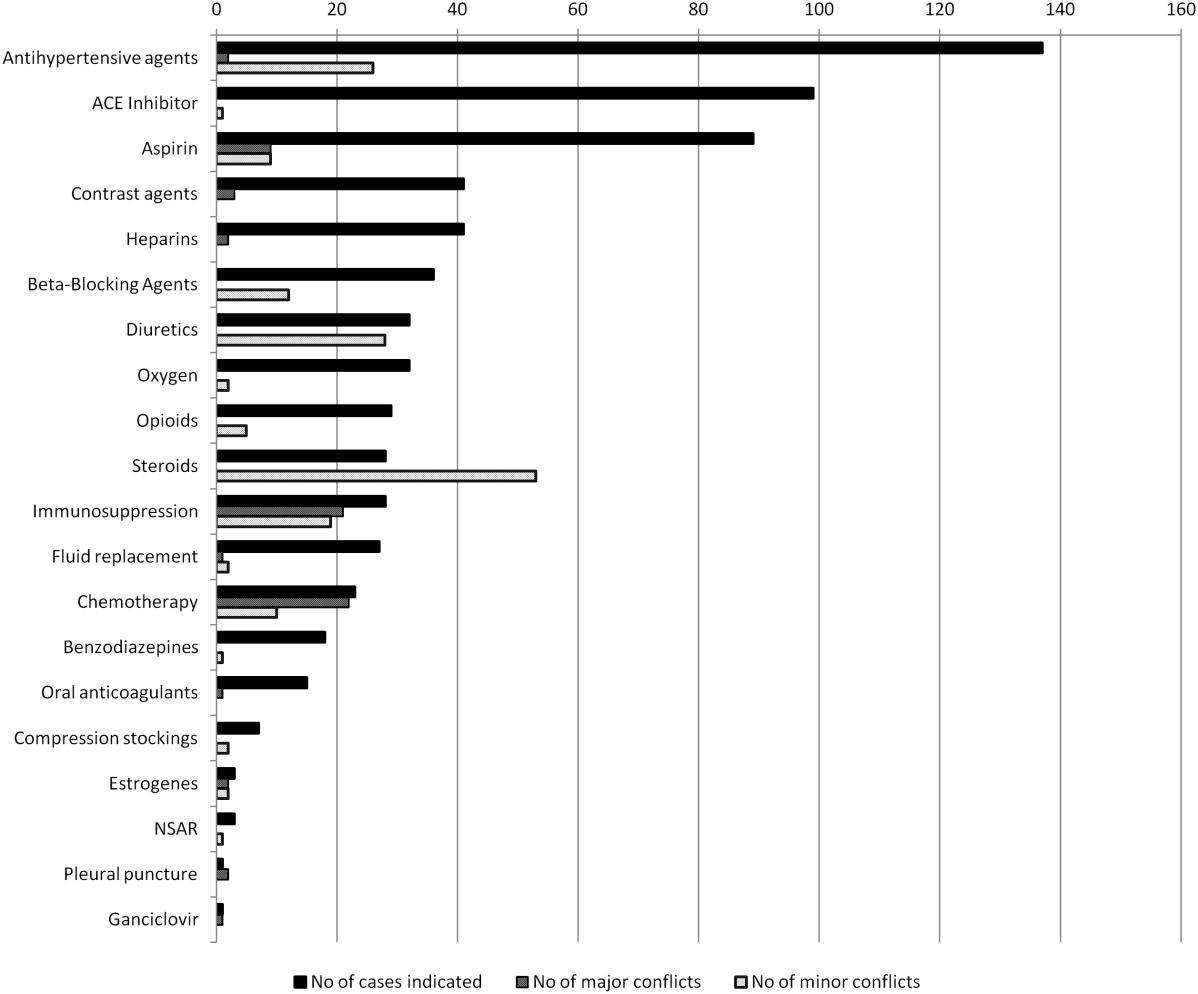
Number of patients with recommended therapies and the associated therapeutic conflicts identified in 166 emergency patients with multimorbity*. ACE = angiotensin-converting enzyme; NSAID = nonsteroidal anti-inflammatory drug *Major therapeutic conflict was defined as a situation where clinical practice guidelines recommend a treatment of one medical condition that is absolutely contraindicated because of a co-existing condition. *Minor* therapeutic conflict was defined as a where clinical practice guidelines recommend a treatment of one medical condition that is relatively contraindicated because of a co-existing condition, but where the treatment is possible without adverse effects if certain precautions are taken.

Figure 2 shows the therapies recommended by clinical practice guidelines that were associated with therapeutic conflicts. From the therapies indicated in more than 10% of the patients, corticosteroids caused the highest number of therapeutic conflicts per patient (indicated in 14% of the patients, caused 1.89 therapeutic conflicts per patient with this indication), followed by immunosuppression (indicated in 17% of the patients, caused 1.4 therapeutic conflict per patient with this indication) and chemotherapy (indicated in 14% of the patients, caused 1.39 therapeutic conflict per patient with this indication).

**Figure 3:**
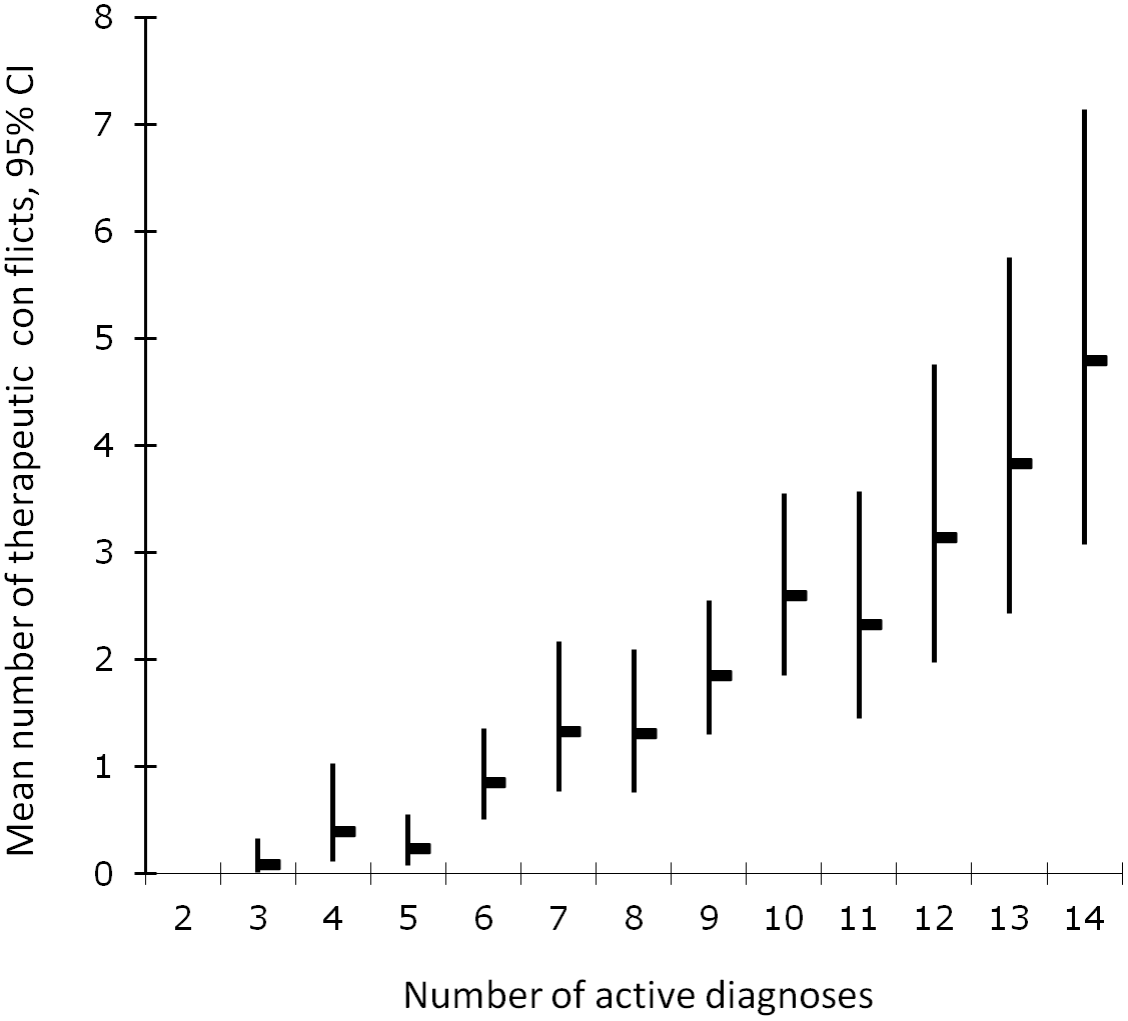
Mean number of therapeutic conflicts with respect to the number of concurrent medical conditions per patient in 166 emergency patients with multimorbity. CI = confidence interval.

The number of therapeutic conflicts was significantly associated with the number of active diagnoses per patient. On average, the number of conflicts increased by 23% for every additional diagnosis (Poisson, p<0.0005). The mean number of conflicts per patient increased uniformly with the number of diagnoses. Figure 3 shows the association between the number of active diagnoses and the mean number of therapeutic conflicts per patients.

## Discussion

In this study, we identified at least one therapeutic conflict in every second patient admitted to the emergency department and subsequently to a hospital medical ward. Major therapeutic conflicts were identified in every third patient. The most commonly occurring major conflicts were in patients with an acute infectious disease who were simultaneously undergoing immunosuppressive therapy or chemotherapy. This constellation of conditions and therapies accounted for almost half of the major conflicts identified in the study. Cytopenia in patients with immunosuppressive therapy or chemotherapy and acute bleeding in patients who required anticoagulation or antiplatelet medication for an underlying cardiovascular disease were each found in one fifth of all major therapeutic conflicts. The most important source of minor therapeutic conflicts was corticosteroids because of their potential for adversely affecting several chronic conditions such as hypertension, diabetes, and osteoporosis.

Antihypertensive therapy was by far the most frequently inidcated therapy (in more than 80% of the patients) overall in the study. Mostly, it was indicated because of primary hypertension, but also in patients with cerebrovascular disease and other vascular diseases without primary arterial hypertension mentioned in their diagnosis list. Nevertheless, antihypertensive therapy generated only 28 therapeutic conflicts (e.g., when indicated in situations with co-existing gastrointestinal bleeding, sepsis, or orthostatic dysregulation). This suggests that continuing antihypertensive therapy is feasible in most situations because the broad spectrum of antihypertensive substances available often makes it possible to avoid conflicts by selecting an appropriate alternative medication. Similarly, in renal failure we found that most conflicts might be avoided by reducing the dose of the medication or by using an alternative medication. Also, most drug allergies we encountered rarely led to therapeutic conflicts, because equivalent alternative therapies were available in most cases.

Previous research has used predefined lists or computer programs to screen for potentially harmful drug-disease combinations. Several lists of inappropriate or potentially harmful drug-disease combinations have been published to date [18]–[22], which several authors have used for estimating the prevalence of potentially harmful drug-disease combinations in hospitalized patients, a number that has ranged from 21% to 51% of the hospitalized population. [23]–[28] In contrast to our study, however, drugs and therapies that were analyzed in previous studies were actually administered to patients. Whether these drugs were used by mistake, by uncritically following guidelines or whether attending physicians were taking calculated risks in these cases remains unknown. In our study we did not measure the incidence of potentially inappropriate prescriptions but illustrated how potentially harmful prescriptions can emanate form clinical practice guidelines. Thus our study also reveals a barrier to applying clinical practice guidelines in daily clinical routine. As we did not follow previously established criteria for potentially inappropriate prescribing, we may have covered a broader spectrum of potential therapy-disease interactions not covered by predefined criteria e.g. by detecting more rarely encountered therapeutic conflicts as well as therapeutic conflict caused by non-pharmacological interventions such as pleurocentesis or compression stockings.

### Limitations

More than 20% of the patients in our study population had an active malignancy; more than 10% of patients were organ transplant recipients. Many of the major therapeutic conflicts that we identified were associated with these medical conditions. Thus, our findings cannot be generalized to apply to hospitals that do not provide specialized services for those medical conditions. Nevertheless, we did identify other therapeutic conflicts that are likely to be encountered in less specialized emergency departments, such as indications for corticosteroid therapy in patients with diabetes or indications for anticoagulation medication in bleeding patients.

We cannot exclude the possibility that our approach to classifying therapeutic conflicts may have biased our results to a higher or lower rate of therapeutic conflicts. However, our finding of therapeutic conflicts was similar to other studies that report potential drug-disease interactions in similar settings [24], [26], [27].

In this pilot study, we did not investigate how therapeutic conflicts were actually dealt with at the time of the patient’s treatment in the emergency department and on the medical ward, nor did we analyze whether the identified therapeutic conflicts were relevant in terms of decisions about further clinical management.

### Conclusions

Therapeutic conflicts are common in multimorbid inpatients. This can severely limit the applicability of clinical practice guidelines because the recommendations for treating the respective conditions are in conflict. Although clinical guidelines cannot address every potential therapeutic conflict, guidance should be available for the most frequently encountered situations in which these conflicts occur, especially for situations where the potential for an adverse outcome is great when therapeutic issues are not addressed.

## Supporting Information

Table S1 **Example of a spreadsheet on an individual patient (Checking applicability of treatment recommendations).**
(DOCX)
