# Supplementary material for: Therapeutic Conflicts in Emergency Department Patients with Multimorbidity: A Cross-Sectional Study
Source: PLoS One. 2014 Oct 13;9(10):e110309. doi: 10.1371/journal.pone.0110309 (PMC4195608; doi:10.1371/journal.pone.0110309)
Supplement: Table S1 — Example of a spreadsheet on an individual patient (Checking applicability of treatment recommendations). (DOCX) [file pone.0110309.s001.docx]

|  |  | Recommended therapies | | | | | | | |
| --- | --- | --- | --- | --- | --- | --- | --- | --- | --- |
|  | Patient number = 134  Sex = male Age = 83 Number of minor conflicts ("1") = 2 Number of major conflicts ("2") = 1 | Search focus, consider empiric treatment | Acetylsalicylic acid, Statin | Identify cause of anemia | Consider calcium acetate, vitamin D and Cinacalcet | Vitamins | Antihypertensive Medication | Immunosuppresion / Corticosteroids | Control blood pressure with ACE inhibitor |
|  |  |  |  |  |  |  |  |  |  |
|  |  |  |  |  |  |  |  |  |  |
|  |  |  |  |  |  |  |  |  |  |
|  |  |  |  |  |  |  |  |  |  |
|  |  |  |  |  |  |  |  |  |  |
|  |  |  |  |  |  |  |  |  |  |
| Diagnoses | Infection with unknown focus |  |  |  |  |  |  | 2 |  |
|  | Occlusive cerebrovascular disease |  |  |  |  |  |  |  |  |
|  | Normocytic, hypochromic anemia (Hb 12.2 g/dL) |  |  |  |  |  |  |  |  |
|  | Secondary hyperparathyroidism |  |  |  |  |  |  |  |  |
|  | Addiction to alcohol |  |  |  |  |  |  |  |  |
|  | Arterial hypertension |  |  |  |  |  |  | 1 |  |
|  | Microscopic polyangiitis |  |  |  |  |  |  |  |  |
|  | Chronic renal failure (GFR 27mL/min) |  |  |  |  |  |  | 1 |  |
